# A Rare Cause of Postpartum Low Back Pain: Pregnancy- and Lactation-Associated Osteoporosis

**DOI:** 10.1155/2014/287832

**Published:** 2014-11-30

**Authors:** Rabia Terzi, Hasan Terzi, Tülay Özer, Ahmet Kale

**Affiliations:** ^1^Department of Physical Medicine and Rehabilitation, Kocaeli Derince Education and Research Hospital, Derince, Kocaeli 41000, Turkey; ^2^Department of Obstetrics and Gynecology, Kocaeli Derince Education and Research Hospital, Derince, Kocaeli 41000, Turkey; ^3^Department of Radiology, Kocaeli Derince Education and Research Hospital, Derince, Kocaeli 41000, Turkey

## Abstract

Pregnancy- and lactation-associated osteoporosis (PLO) is a rare form of osteoporosis. It results in severe low back pain in the last trimester of pregnancy and in the postpartum period, decreases in height, and fragility fractures, particularly in the vertebra. The current case report presents a 32-year-old patient who presented with back and low back pain that began in the last trimester of the pregnancy and worsened at two months postpartum and who was diagnosed with pregnancy- and lactation-associated osteoporosis after exclusion of other causes; the findings are discussed in view of the current literature. PLO is a rare clinical condition causing significant disability. PLO must be kept in mind in the differential diagnosis in patients presenting with low back pain during or after pregnancy. The patients must be evaluated for the risk factors of PLO, and an appropriate therapy must be initiated.

## 1. Introduction

PLO is a rare condition affecting pregnant or breastfeeding women and it is an important type of osteoporosis causing a significant morbidity [1]. The incidence of PLO is 0.4 in 100,000 women. It is considered that the number of undiagnosed patients is even higher [2]. Although its etiology is unclear, the presence of PLO in first degree relatives, low BMI, physical inactivity, poor nutrition, insufficient calcium intake, and smoking have been determined as risk factors [3, 4]. The patients present with severe low back pain in the last trimester of the pregnancy or in the postpartum period or height decrease secondary to fragility fractures in the vertebra [5]. Pregnancy- and lactation-associated osteoporosis is often confused with other causes of low back pain during pregnancy, and it is therefore important to keep this in mind in the differential diagnosis. This case report presents a patient who presented with low back pain and spinal vertebra fractures in the postpartum period after her first pregnancy and who was diagnosed with pregnancy- and lactation-associated osteoporosis; the findings are discussed in light of the current literature.

## 2. Case Presentation

**Figure 1:**
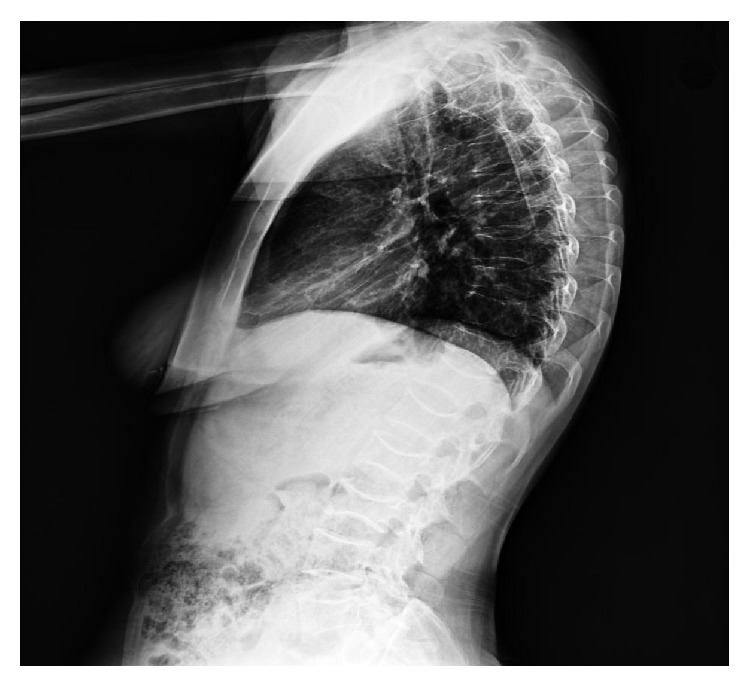
Lateral thoracolumbar X-ray; loss of height in vertebrae and biconcave appearance in vertebrae.

**Figure 2:**
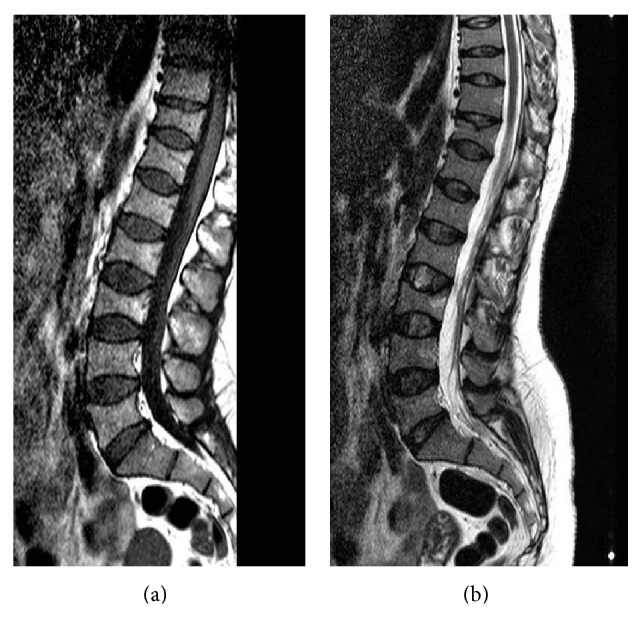
Lumbar MRI de T1 (a) and T2 (b); loss of height in multiple vertebral bodies in various degrees and a biconcave appearance in vertebral bodies.

A 32-year-old female patient was referred to our clinic due to back and low back pain that occurred at two months postpartum. The patient's medical history was not remarkable for chronic disease, drug use, smoking, or alcohol use. The patient reported that the pain started one month before delivery and gradually worsened in the postpartum period. She noticed a height decrease during pregnancy from her clothes fitting more loosely. On the physical examination, the patient was mobile with the aid of forearm crutches. The range of motion in thoracic and lumbar joints was restricted and painful, and there was tenderness with palpation on spinal processes in the thoracolumbar region. There was also paravertebral muscle spasm, and thoracic kyphosis was minimally increased. The pain level was 9 on the VAS. Lumbosacral radiography and MRI showed a loss of height in multiple vertebral bodies in various degrees and a biconcave appearance in vertebral bodies (Figures 1 and 2). Dual energy X-ray absorptiometry (DEXA) revealed a lumbar *Z*-score of −3.2 and a femoral *Z*-score of −1.8. The liver and kidney function tests, erythrocyte sedimentation rate, C-reactive protein, thyroid function tests, calcium, phosphorus, alkaline phosphatase, parathyroid hormone, osteocalcin, and *β* CTX levels, and protein electrophoresis revealed normal findings, and 25-OH vitamin D levels were slightly decreased (21.3 ng/mL). The patient was diagnosed with PLO, and an appropriate treatment was initiated. The patient was primarily recommended to discontinue breastfeeding and maintain a balanced diet. The patient was administered with calcium (1200 mg/day) and vitamin D supplementation (800 IU/day) and antiresorptive therapy (alendronate 70 mg/week). For pain management, analgesics and physical therapy involving transcutaneous electrical nerve stimulation, hot-pack application, postural and muscle strengthening exercises, and thoracolumbar corset were recommended to the patient. A significant reduction was achieved in pain scores during the visit at two weeks. The pain score was 3 in the VAS. The patient was placed on follow-up.

## 3. Discussion

The pregnancy and lactation periods are characterized by dynamic changes occurring in bone metabolism and musculoskeletal pain caused by biomechanical and hormonal factors [6]. The thorough knowledge of the pathological changes occurring during these periods is particularly important in early diagnosis and treatment.

PLO is a rare form of osteoporosis affecting primarily primiparous women. The clinical picture may involve severe and persistent back and low back pain to the extent that the women may have difficulty in holding the baby and height decrease associated with vertebral fragility fractures [7]. In the literature, a case of PLO was reported that sustained fractures in eight vertebrae [8]. Although not specified as a diagnostic criterion, the exclusion of other reasons for osteoporosis and progressive clinical course are helpful in the diagnostic process [5]. The medical history of some patients reported to have PLO in the literature was remarkable for secondary causes of osteoporosis such as oligomenorrhoea, infertility therapy with clomiphene, gluten enteropathy, and heparin administration [9, 10]. The current case did not have any condition that could result in secondary osteoporosis. The identified risk factors were low BMI and insufficient calcium intake.

The radiological imaging in such patients is helpful in detecting osteoporosis and fractures. DEXA shows osteoporosis [5]. Spinal bone mineral density (BMD) in these patients was lower than femoral BMD [4, 9]. Spinal BMD was also lower in the present case.

The aim of therapy is to prevent new fractures, increase BMD, and prevent the development of chronic pain. The cessation of breastfeeding is recommended in most cases [11]. This condition is not considered to be a contraindication for subsequent pregnancy [5]. There is no mutually agreed opinion or guideline in the treatment of this condition. The treatment options are limited to those practiced in the reported cases. These options include calcium and vitamin D [11], bisphosphonates [4], teriparatide [12], and strontium ranelate [13]. In the literature, there are reports of patients who underwent a kyphoplasty procedure for vertebral fractures [14]. The current case was administered with calcium and vitamin D supplementation, weekly bisphosphonates, and pain therapy. Studies have demonstrated that bisphosphonates provide significant improvement in clinical findings and BMD values in patients with PLO [2, 4]. Bisphosphonates, calcium, and vitamin D supplementation are regarded as the first line of therapy in the treatment of PLO [5]. However, long-term safety studies are warranted due to the fact that prenatal side effects of bisphosphonates are not known in premenopausal, breastfeeding women and in those intending subsequent pregnancy and due to accumulation of the drug in the bones [15, 16].

PLO is a rare clinical entity, and it must be kept in mind in the differential diagnosis in patients presenting with low back pain during and after pregnancy. The fractures related to PLO may have a negative impact on the relationship between the mother and the baby due to restrictions in daily activities and associated pain, and this condition is an important cause of disability in the long term. Early diagnosis and treatment of these cases are particularly important in the prevention of fractures and increasing the quality of life of the patients.
